# Utility of magnetic resonance imaging for differentiating malignant mesenchymal tumors of the uterus from T2-weighted hyperintense leiomyomas

**DOI:** 10.1007/s11604-021-01217-2

**Published:** 2021-11-09

**Authors:** Koichiro Matsuura, Kaiji Inoue, Eri Hoshino, Masanori Yasuda, Kosei Hasegawa, Yoshitaka Okada, Yasutaka Baba, Eito Kozawa

**Affiliations:** 1 Department of Radiology, Saitama Medical University, 38, Morohongo, Moroyamamachi, Saitama Japan; 2 Department of Pathology, Saitama Medical University, 38, Morohongo, Moroyamamachi, Saitama Japan; 3 Department of Gynecologic Oncology, Saitama Medical University, 38, Morohongo, Moroyamamachi, Saitama Japan; 4 Department of Diagnostic Radiology, Saitama Medical University International Medical Center, 1397-1 Yamane, Hidaka, Saitama Japan

**Keywords:** Leiomyosarcoma, Endometrial stromal sarcoma, Undifferentiated uterine sarcoma, Leiomyoma, MRI

## Abstract

**Purpose:**

To generate a new discrimination method to distinguish between malignant mesenchymal tumors of the uterus and T2-weighted hyperintense leiomyoma based on magnetic resonance imaging findings and clinical features.

**Materials and methods:**

Data from 32 tumors of 32 patients with malignant mesenchymal tumors of the uterus and from 34 tumors of 30 patients with T2-weighted hyperintense leiomyoma were analyzed. Clinical parameters, qualitative magnetic resonance imaging features, including computed diffusion-weighted imaging, and quantitative characteristics of magnetic resonance imaging of these two tumor types were compared. Predictive values for malignant mesenchymal tumors of the uterus were calculated using variant discriminant analysis.

**Results:**

The T1 bright area on qualitative assessment and mean apparent diffusion coefficient value on quantitative assessment yielded the most independent magnetic resonance imaging differentiators of malignant mesenchymal tumors of the uterus and T2-weighted hyperintense leiomyoma. The classification accuracy of the variant discriminant analysis based on three selected findings, i.e., a T1 bright area, computed diffusion-weighted imaging with a b-value of 2000s/mm^2^ (cDWI_2000_), and T2-hypointense bands, was 84.8% (56/66), indicating high accuracy.

**Conclusions:**

Variant discriminant analysis using the T1 bright area, cDWI_2000_, and T2-hypointense bands yielded high accuracy for differentiating between malignant mesenchymal tumors of the uterus and T2-weighted hyperintense leiomyoma.

## Introduction

Malignant mesenchymal tumors of the uterus (MMTUs), such as leiomyosarcoma, endometrial stromal sarcoma, undifferentiated uterine sarcoma, and smooth muscle tumor of uncertain malignant potential, are a rare heterogeneous group of malignant tumors of mesenchymal origin with poor prognosis [1–4]. Hysterectomy and bilateral oophorectomy are required for treating MMTUs [2–4]. In contrast, leiomyomas of benign mesenchymal tumors are the most common uterine neoplasms [1, 4–7]. For symptomatic leiomyoma treatment, minimally invasive surgery is needed to reduce postoperative pain, shorten hospitalization, and preserve fertility [4–7]. Therefore, accurate differentiation of malignant and benign mesenchymal tumors is critical for selecting the proper treatment.

Magnetic resonance imaging (MRI) is the most important diagnostic tool for differentiating between uterine leiomyoma and sarcoma [3, 8–18]. However, single-image findings have limited distinguishing ability and are not considered sufficiently sensitive or specific, such as signs of hemorrhage, tumor margins, and apparent diffusion coefficient (ADC) values [3, 8–18]. Misdiagnosis or delayed diagnosis of MMTU is not uncommon in clinical practice, and one of the reasons is the similarity of MRI findings between MMTUs and uterine leiomyomas. For example, high signal intensity on T2-weighted images (T2WI) is one of the characteristics of MMTUs; however, uterine leiomyomas may also show hyperintensity due to degeneration, edema, and increased cellularity [19–21]. Therefore, it is difficult to distinguish between MMTUs and uterine leiomyomas that show a signal intensity higher than that indicated by the myometrium on T2WI, and it is clinically very important to differentiate them. Several studies have attempted to create a diagnostic method to distinguish between MMTUs and benign leiomyomas using multivariate analysis; however, they included low-signal leiomyomas on T2WI and had a limited sample size [9, 14, 22]. This study aimed to generate a new method to distinguish MMTUs and uterine leiomyomas wherein more than 50% of the lesions show higher signal intensity than that revealed by the myometrium on T2WI (T2-weighted hyperintense leiomyoma: T2HILM) based on the findings of MRI and clinical features.

## Materials and methods

### Patient population

Our Institutional Review Board approved this retrospective study, and informed consent was obtained in the form of an opt-out on the website.

One author with 30 years of experience retrospectively searched for eligible patients in our institutional pathological and MRI databases between May 2007 and August 2020. The inclusion and exclusion criteria were as follows: (1) patients with MMTU, cellular leiomyoma, and degenerative leiomyoma confirmed through pathology were included; (2) patients with tumors comprising more than 50% of areas of signal intensity higher than that of the myometrium on T2WI were included; (3) patients who underwent surgery and had complete MRI data within 3 months before surgery were included; (4) patients who were pregnant or with histories of preoperative chemotherapy, radiotherapy, or hormonal therapy were excluded; and (5) patients with carcinosarcoma were excluded according to the WHO Classification of Tumours. Female Genital Tumours, revised in the 5th edition [1].

The final study population consisted of 62 patients with surgically resected and pathologically proven MMTU (32 patients; 32 tumors; age range, 39–74 years; mean age, 56 years) or T2HILM (30 patients; 34 tumors; age range, 31–82 years; mean age, 49 years). The distribution of the pathological results is shown in Table 1.

**Table 1:** Pathological diagnosis

Pathologic results	Number of cases
*MMTU*	32
Leiomyosarcoma	16
Endometrial stromal sarcoma (low grade)	7
Endometrial stromal sarcoma (high grade)	5
Undifferentiated uterine sarcoma	2
STUMP	2
*T2HILM*	34
Cellular leiomyoma	15
Hyaline degeneration	4
Hydropic degeneration	2
Mucinous degeneration	1
Cystic degeneration	1
Mixed degeneration	11

*MMTU* malignant mesenchymal tumors of uterus, *STUMP* smooth muscle tumor of uncertain malignant potential, *T2HILM* T2-weighted hyperintense leiomyoma

### Clinical parameters

Information regarding age and abnormal vaginal bleeding was collected for patients with MMTU and T2HILM.

### MRI protocol

Pelvic MRI was performed using a 1.5-T scanner (MAGNETOM Avanto 1.5 T, Siemens, Erlangen, Germany; or Nova Dual 1.5 T, Philips Healthcare, The Netherlands) and 3.0-T MR superconducting units (Intera Achieva 3.0 T; Philips Healthcare, Best, The Netherlands) with a phased-array multicoil. T1WI and T2WI in the oblique sagittal section were obtained along the long axis of the uterus, and T2WI and diffusion-WI (DWI) in the transverse axial section were obtained. A three-dimensional fat-suppressed contrast-enhanced T1WI protocol was included in the MRI. Before MRI, 20 mg of butyl scopolamine (Buscopan, Boehringer Ingelheim, Ingelheim am Rhein, Germany) was intramuscularly injected to suppress bowel peristalsis. The MRI protocols are summarized in Table 2.

**Table 2:** Scan parameters of magnetic resonance imaging

Parameters	T1WI		T2WI		FsGdT1WI		DWI	
	1.5 T	3.0 T	1.5 T	3.0 T	1.5 T	3.0 T	1.5 T	3.0 T
TR (ms)	200–500	150–600	2500–5600	3500–5600	3–690	3–700	3200–6200	6000–11,250
TE (ms)	2–12	1–11	80–105	90–100	1–12	2–9	70–80	70–75
FOV (cm)	28 × 32	28 × 33	28 × 35	28 × 36	28 × 38	28 × 39	32 × 35	32 × 36
Slice thickness (mm)	5–6	4–5	5–6	4–5	4–6	2–5	5–6	4–5
*b* value (mm^2^/s)	–	–	–	–	–	–	0, 500, 1000	0, 500, 1000

*T1WI* T1-weighted image, *T2WI* T2-weighted image, *FsGdT1WI* post-contrast fat-saturated T1WI, *DWI* diffusion-weighted image, *TR* repetition time, *TE* echo time, *FOV* fields of view

### Data processing and image interpretation

ADC maps were generated automatically from each DWI (*b*-values = 0, 500, and 1000 s/mm^2^) using the MR system software. ADC histograms of every tumor slice were generated, and the mean ADC value, skewness, and kurtosis were calculated using commercially available software (SYNAPSE VINCENT version 4.4, Fujifilm, Tokyo, Japan). Computed diffusion-weighted imaging (cDWIs) with *b*-values of 1500 s/mm^2^ (cDWI_1500_), 2000s/mm^2^ (cDWI_2000_), 2500 s/mm^2^ (cDWI_2500_), 3000 s/mm^2^ (cDWI_3000_), 3500 s/mm^2^ (cDWI_3500_), and 4000 s/mm^2^ (cDWI_4000_) were generated from real measured DWIs (rDWI) with *b*-values of 0, 500, and 1000 s/mm^2^ by fitting a mono-exponential model using SYNAPSE VINCENT software.

**Fig. 1:**
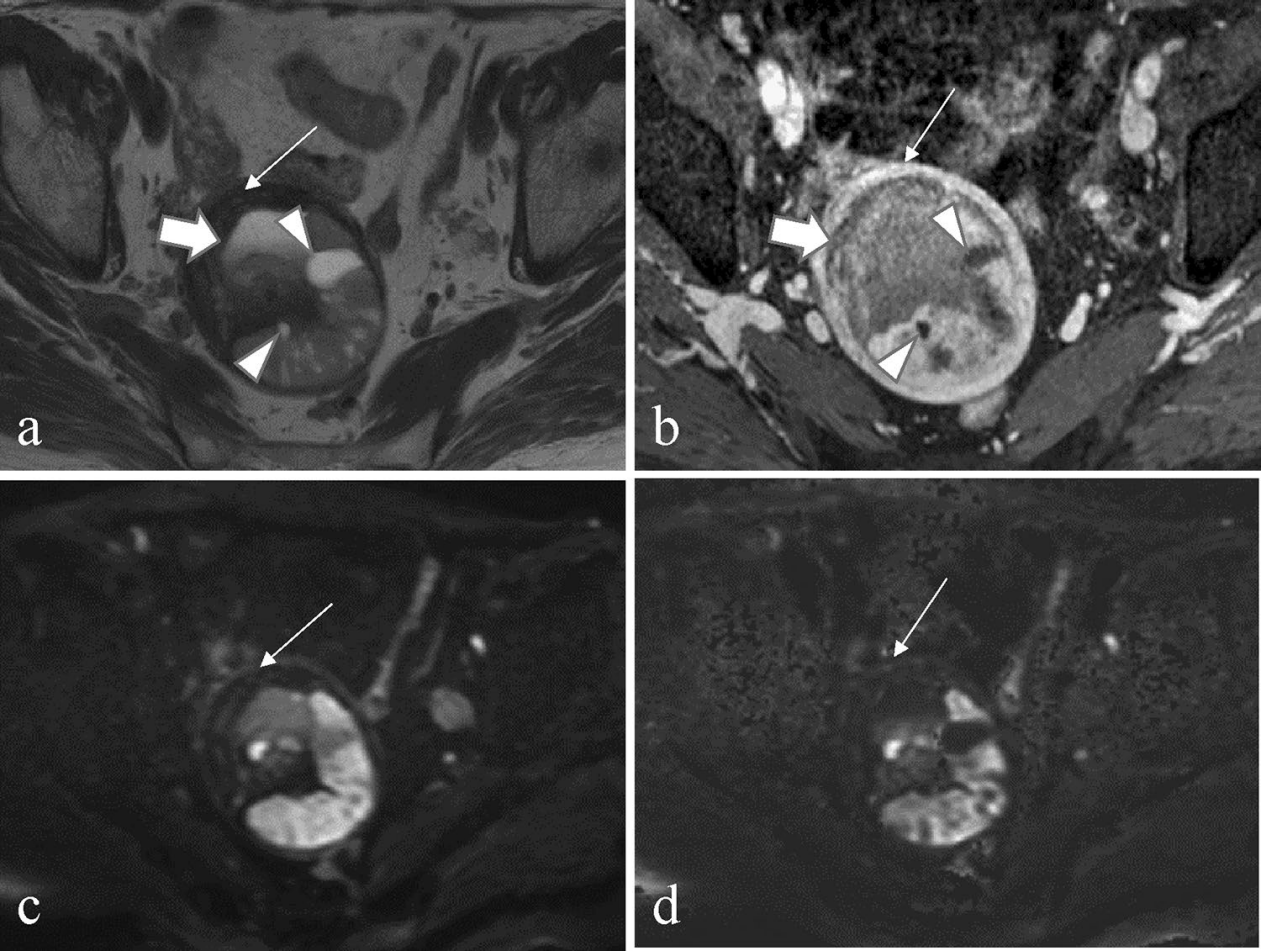
A 76-year-old woman with endometrial stromal sarcoma without abnormal vaginal bleeding. **a** The axial T2-weighted image and **b** axial contrast-enhanced T1-weighted image with fat suppression show a tumor, located mainly in the myometrium, with hemorrhagic necrosis (arrow: necrosis and T2 dark area) and cyst (arrowhead). **c** On an axial diffusion-weighted image with a *b*-value of 1000 s/mm^2^ and **d** an axial computed diffusion-weighted image with a *b*-value of 2000s/mm^2^, the solid component of endometrial stromal sarcoma appears to have a higher signal than the myometrium (thin arrow). The lesion shows restricted diffusion, and the mean apparent diffusion coefficient value is 0.86 × 10^−3^ mm^2^/s

**Fig. 2:**
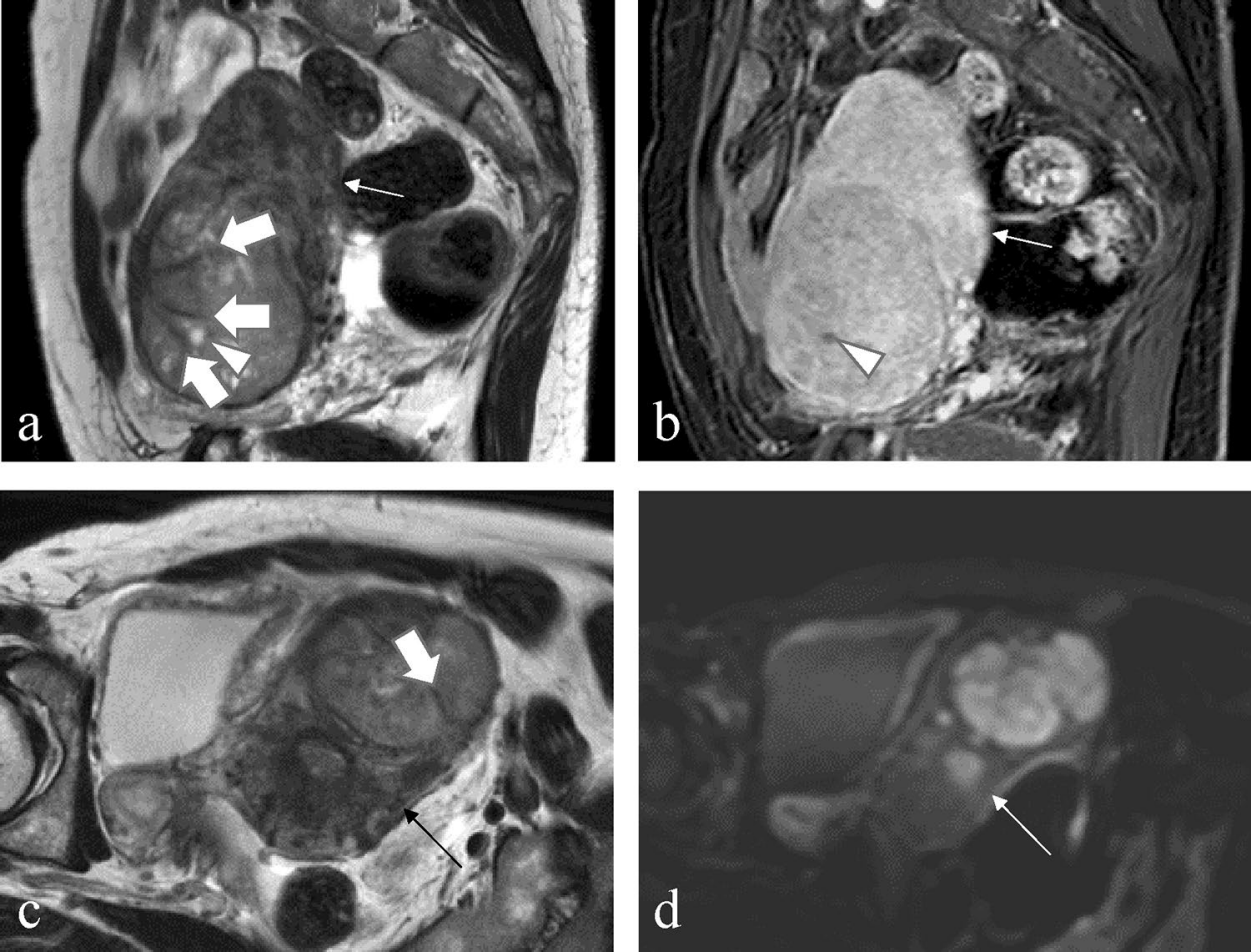
A 48-year-old woman with endometrial stromal sarcoma without abnormal vaginal bleeding. **a** The sagittal T2-weighted image and **b** the sagittal contrast-enhanced T1-weighted image with fat suppression reveals a tumor, locates mainly in the myometrium, with cyst (arrowhead). **a** The sagittal T2-weighted image and **c** the axial T2-weighted image indicates the lesion with T2-hypointense bands (arrow). **d** On an axial diffusion-weighted image with a *b*-value of 1000 s/mm^2^, the tumor showed a higher signal than the myometrium (thin arrow). The lesion shows restricted diffusion, and the mean apparent diffusion coefficient value is 1.03 × 10^−3^ mm^2^/s

**Fig. 3:**
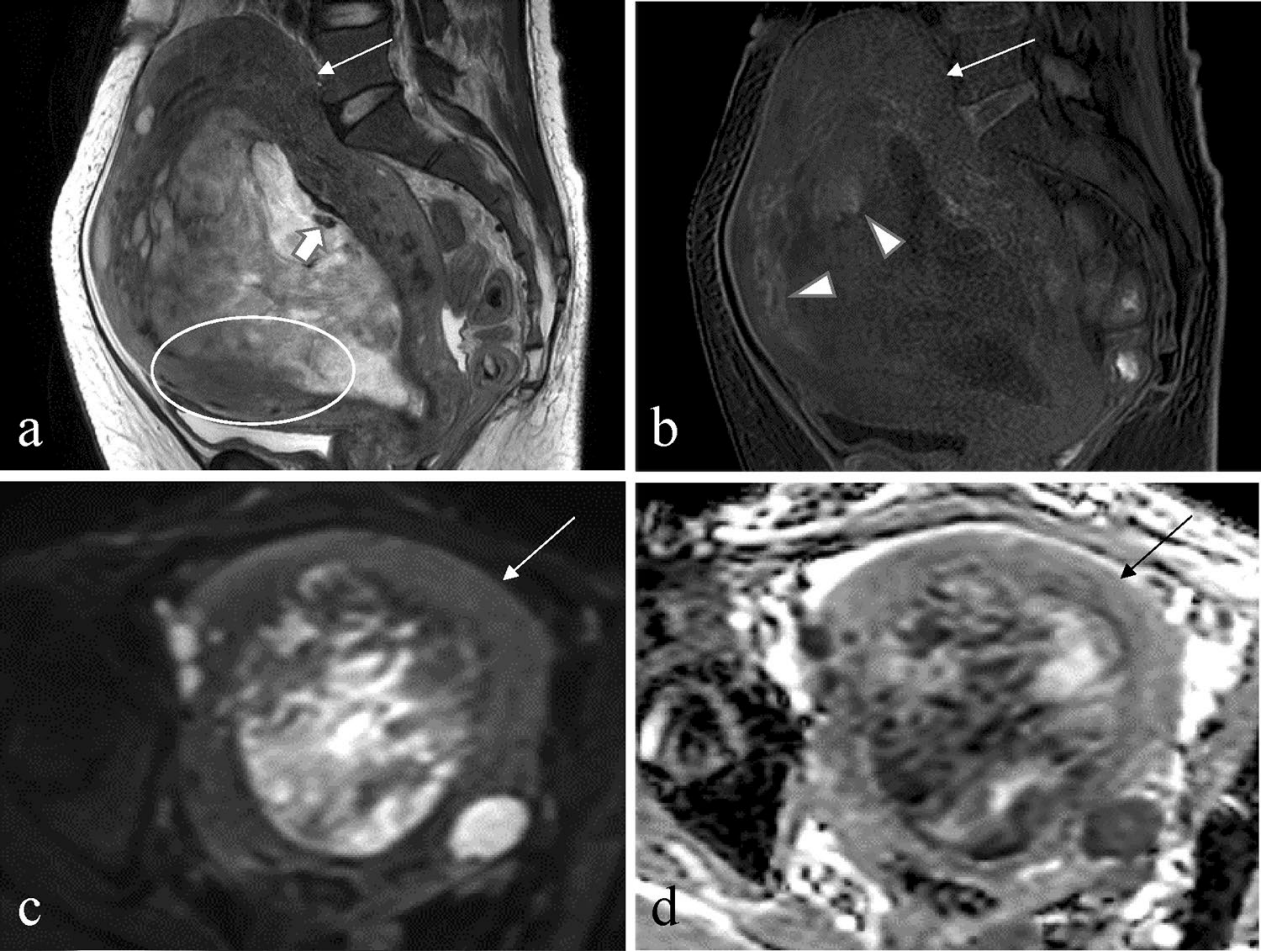
A 56-year-old woman with leiomyosarcoma with abnormal vaginal bleeding. **a** The sagittal T2-weighted image shows a tumor, mainly located in the uterine cavity, with an ill-defined margin (circle) and T2 dark area (arrow). The tumor shows signal heterogeneity on the T2-weighted image. **b** The tumor shows a T1 bright area (arrowhead) on the sagittal T1-weighted image. **c** On an axial diffusion-weighted image with a *b*-value of 1000 s/mm^2^, the lesion appears to have a higher signal than the myometrium (thin arrow). **d** The axial apparent diffusion coefficient map reveals that the solid component of leiomyosarcoma shows restricted diffusion, and the mean apparent diffusion coefficient value is 0.77 × 10^−3^ mm^2^/s

**Fig. 4:**
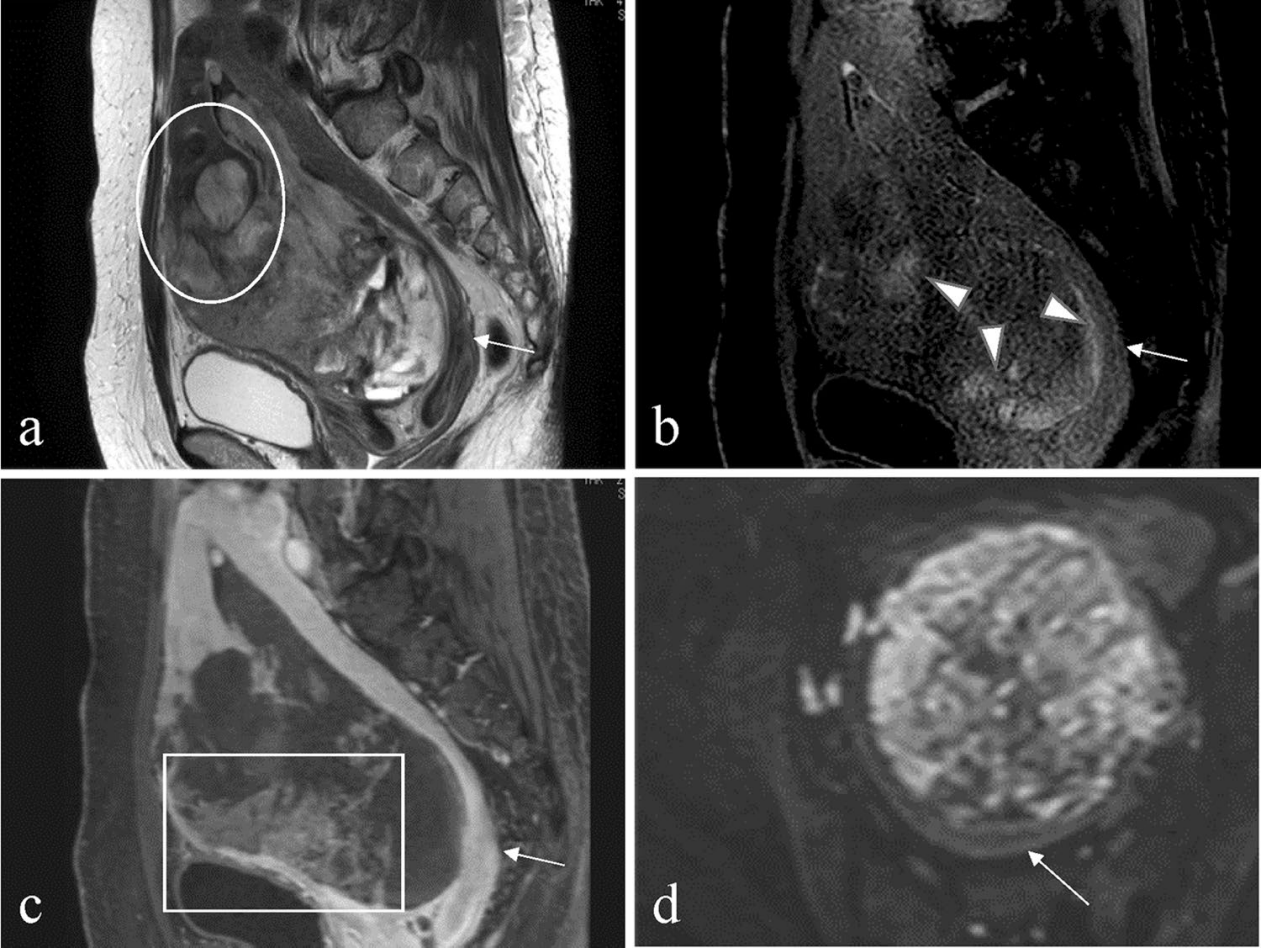
A 49-year-old woman with undifferentiated uterine sarcoma with abnormal vaginal bleeding. **a** The sagittal T2-weighted image reveals a tumor located mainly in the uterine cavity, with a nodular border (circle). The tumor shows a heterogeneous signal on the T2-weighted image. **b** The tumor shows T1 bright area (arrowhead) on the sagittal T1-weighted image. **c** The contrast-enhanced T1-weighted image with fat suppression indicates a lesion with feather-like enhancement (rectangle). **d** On an axial diffusion-weighted image with a *b*-value of 1000 s/mm^2^, the solid component of undifferentiated uterine sarcoma shows a higher signal than the myometrium (thin arrow). The lesion shows restricted diffusion, and the mean apparent diffusion coefficient value is 0.90 × 10^−3^ mm^2^/s

**Fig. 5:**
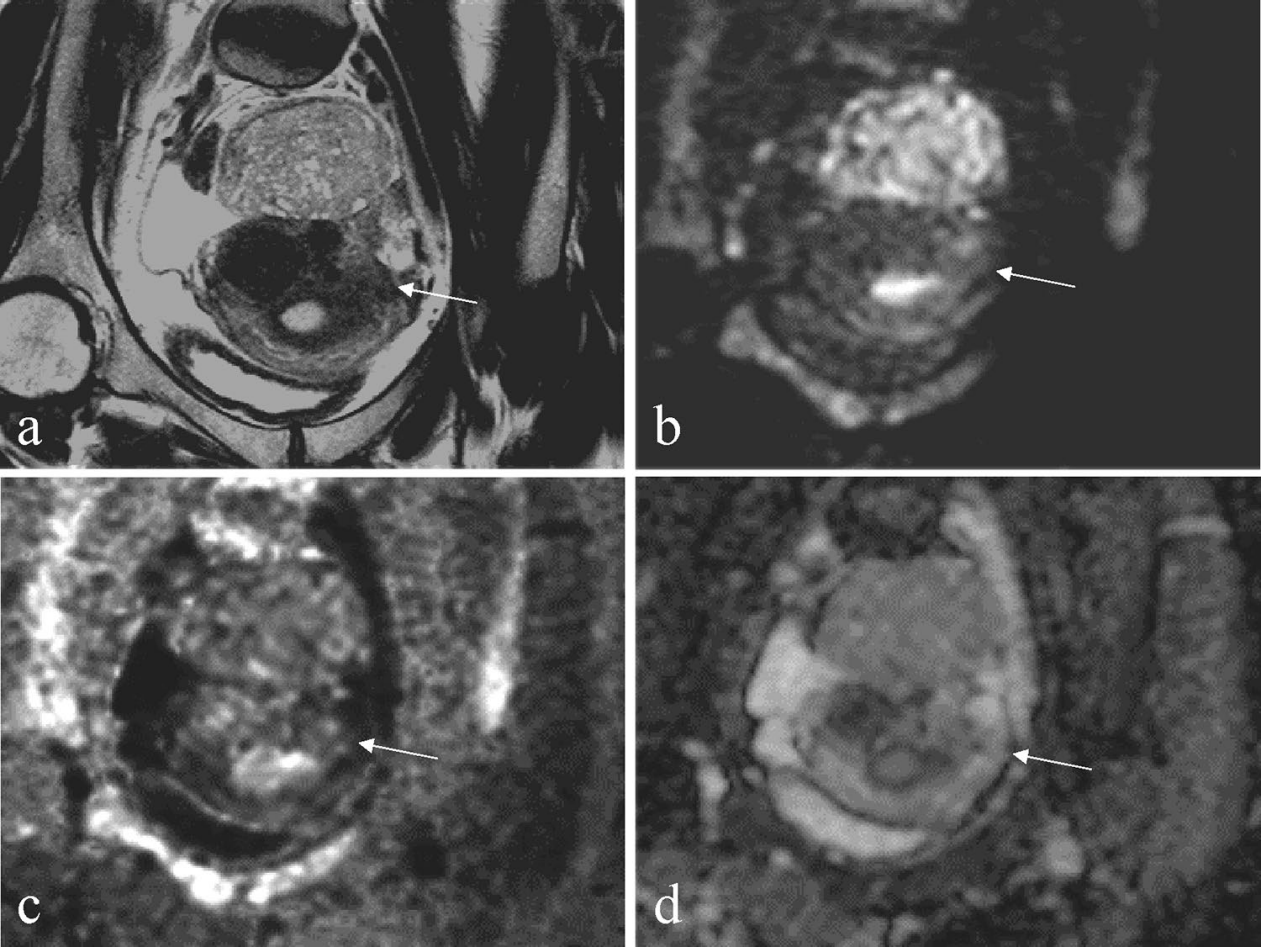
A 42-year-old woman with cellular leiomyoma without abnormal vaginal bleeding. **a** The coronal T2-weighted image reveals a tumor locates mainly in the subserosal location. The tumor shows a higher signal intensity than the myometrium on a T2-weighted image. **b** On a coronal diffusion-weighted image with a *b*-value of 1000 s/mm^2^, the lesion shows a higher signal than the myometrium (thin arrow). However, **c** the signal was equal to that of the myometrium on a coronal computed diffusion-weighted image with a *b*-value of 2000s/mm^2^. **d** Using the coronal apparent diffusion coefficient map, the lesion is determined to have a mean apparent diffusion coefficient value of 1.65 × 10^−3^ mm^2^/s

Two radiologists with 6 and 4 years of experience, respectively, both blinded to the pathological diagnosis and clinical information, evaluated several MRI features for each index lesion. On qualitative assessment, any discrepancies were resolved by consensus after careful evaluation. Two readers independently assessed the following MRI features: main tumor location, presence of necrosis, cysts, T1 bright area, T2 dark areas, T2-hypointense band, feather-like enhancement, heterogeneity on T2WI, clarity of tumor margin (ill-defined or well-defined), tumor border shape (nodular or smooth), tumor morphology (round or amorphous), presence and location of unenhanced area (central or not), and signal intensity of tumor compared to the myometrium on DWI (rDWI and cDWI). *Necrosis* was defined as an irregular area with high signal intensity on T2WI and lack of enhancement after contrast medium administration (Fig. 1). *Cysts* were defined as well-demarcated areas without enhancement (Figs. 1 and 2). We defined a *T1 bright area* as a signal intensity that was higher or similar to that of the myometrium at pre-enhancement T1WI. It was defined as an indicator of subacute hemorrhage (Figs. 3 and 4). *T2 dark areas* represent areas of chronic hemorrhage that can be observed as a lower signal intensity area than that of the myometrium (Figs. 1 and 3). A *T2-hypointense band* was defined as the band-shaped low-signal area with equal signal to the myometrium observed on T2WI within the lesion (Fig. 2). *Feather-like enhancements* were defined as fine and wispy enhancements interspersed within the tumors [23] as shown in Fig. 4. *Ill-defined margins* were defined as ambiguous and indistinguishable from the adjacent myometrium (Fig. 3). DWI signals were evaluated as shown in Figs. 1, 2, 3, 4 and 5.

For quantitative assessments, two radiologists measured the data, and the data measured by the radiologist with 6 years of experience were used for the analysis. The ADC histogram values (mean ADC value, skewness, and kurtosis) of each primary tumor were measured using manually drawn regions of interest (ROI) on the console with solid components on the largest tumor plane, excluding the necrotic and non-enhanced portions by referring to T1WI, T2WI, and contrast-enhanced images. The largest diameter of the tumor and the non-enhancing area of the tumor (necrosis or cyst) was measured on transverse T2WI and fat-suppressed contrast-enhanced T1WI through the maximum face of the tumor. When there were multiple non-enhancing areas within the same tumor, the largest one was measured. Additionally, the maximum tumor area was measured.

### Statistical analysis

We used several statistical tests to evaluate and analyze the capacity of visual and objective assessments to discriminate between MMTU and T2HILM. Statistical analyses were performed using JMP version 14.0.0 (SAS Institute; Cary, NC, USA). A *p* value < 0.05 was considered statistically significant. Qualitative interobserver agreement was calculated using the Kappa statistic; Kappa scores of 0.0–0.20, 0.21–0.40, 0.41–0.60, 0.61–0.80, and greater than 0.80, indicated no-to-slight, fair, moderate, good, and excellent agreement, respectively [24]. Qualitative assessments between MMTU and T2HILM were analyzed using Fisher’s exact test. Spearman’s correlation coefficient and the intraclass correlation coefficient (ICC) were calculated to test the concordance of interobserver variability. The agreement was interpreted according to the ICC as follows: values less than 0.50, 0.50–0.75, 0.75–0.90, and greater than 0.90 indicated poor, moderate, good, and excellent reliability, respectively [25]. We used the Wilcoxon test to compare the quantitative MRI characteristics. We performed receiver operating characteristic (ROC) curve analyses based on positive test results for patients with MMTU to identify the optimal cutoff for maximizing the sum of sensitivity and specificity. We examined the area under the ROC curve (AUC values of 0.5–0.7, 0.7–0.9, and 0.9–1.0 refer to low, moderate, and high accuracy, respectively) [26] to assess the diagnostic abilities of the largest diameter of the tumor and the non-enhancing part of the tumor, maximum area of the tumor, mean ADC values, skewness, and kurtosis between MMTU and T2HILM.

A variant discriminant analysis was performed to gauge the associations of all qualitative and quantitative values using JMP version 14.0.0 and Multi Tahenryo version 1.1, an Excel add-in software (Institute of Statistical Analyses, Inc., Tokyo, Japan). Quantitative values were classified into two categories based on the AUC cutoff value for a variant discriminant analysis. If the discriminant accuracy rate is 75% or higher or the correlation ratio is 0.5, the relationship equation can be applied to forecasting [27].

## Results

### Qualitative MRI findings of MMTU and T2HILM

The MRI findings of T2HILM differed significantly from those of MMTU, as summarized in Table 3. The interobserver agreement for MRI features was either good or excellent. Particularly, the agreement was excellent for the main location of the tumor component (0.99).

**Table 3:** Qualitative MRI findings and a clinical parameter of T2-weighted hyperintense leiomyoma and MMTU

MRI findings	MMTU	T2HILM	*κ*-value	*p* value
	(*n* = 32)	(*n* = 34)		
Main location of tumor: uterine cavity	7 (21.9%)	4 (11.8%)	0.985	0.333
Necrosis: present	14 (43.8%)	1 (2.9%)	0.873	< .0001^†^
Cyst: present	14 (43.8%)	5 (14.7%)	0.778	0.0139^†^
T1 bright area: present	25 (78.1%)	5 (14.7%)	0.889	< .0001^†^
T2 dark area: present	12 (37.5%)	6 (17.6%)	0.682	0.0983
T2-hypointense band: present	12 (37.5%)	1 (2.9%)	0.822	0.0004^†^
Feather-like enhancement: present	16 (50.0%)	1 (2.9%)	0.855	< .0001^†^
Heterogeneity on T2WI: heterogeneous	28 (87.5%)	29 (85.3%)	0.859	1.000
Clarity of tumor margin: ill-defined	31 (96.9%)	22 (64.7%)	0.680	0.0013^†^
Tumor border shape: nodular	15 (46.9%)	2 (5.9%)	0.818	0.0002^†^
Tumor morphology: amorphous	27 (84.3%)	25 (73.5%)	0.769	0.371
Location of unenhanced area: central	20 (62.5%)	4 (11.8%)	0.869	< .0001^†^
rDWI signal: higher than myometrium	31 (96.9%)	25 (73.5%)	0.890	0.0134^†^
cDWI_1500_ signal: higher than myometrium	31 (96.9%)	23 (67.6%)	0.917	0.0029^†^
cDWI_2000_ signal: higher than myometrium	30 (93.8%)	18 (52.9%)	0.820	0.0002^†^
cDWI_2500_ signal: higher than myometrium	26 (81.3%)	15 (44.1%)	0.738	0.0024^†^
cDWI_3000_ signal: higher than myometrium	23 (71.9%)	8 (23.5%)	0.804	0.0002^†^
cDWI_3500_ signal: higher than myometrium	22 (68.8%)	6 (17.6%)	0.929	< .0001^†^
cDWI_4000_ signal: higher than myometrium	21 (65.6%)	6 (17.6%)	0.964	0.0001^†^
Abnormal vaginal bleeding: present	10 (31.3%)	7 (20.6%)	NA	0.403

*MMTU* malignant mesenchymal tumors of uterus, *T2HILM* T2-weighted hyperintense leiomyoma, *T2WI* T2-weighted image, *rDWI* real measured diffusion-weighted image with *b*-value of 1000 s/mm^2^, *cDWI*_*1500-4000*_ computed diffusion-weighted image (with *b*-values of 1500–4000 s/mm^2^)

^†^Significant difference between MMTU and T2-weighted hyperintense leiomyoma groups

Only one of the T2HILMs demonstrated necrosis, a T2-hypointense band, and feather-like enhancement. In contrast, only one of the MMTUs demonstrated a lower signal than that of the myometrium on rDWI and cDWI_1500_, and one MMTU demonstrated well-defined margins. Necrosis, cysts, T1 bright area, T2-hypointense band, feather-like enhancement, clarity of tumor margin, tumor border shape, location of the unenhanced area, rDWI and cDWI_1500–4000_ showed significant differences (*p* < 0.05). The statistical analysis of each MRI finding is summarized in Table 4.

**Table 4:** Statistical analyses of qualitative MRI findings and a clinical parameter

MRI findings	ACC	Sensitivity	Specificity	PPV	NPV
Main location of tumor	0.561	0.219	0.111	0.179	0.138
Necrosis	0.712	0.438	0.971	0.933	0.647
Cyst	0.652	0.438	0.853	0.737	0.617
T1 bright area	0.818	0.781	0.853	0.833	0.896
T2 dark area	0.606	0.375	0.824	0.667	0.583
T2-hypointense band	0.682	0.375	0.971	0.923	0.623
Feather-like enhancement	0.742	0.500	0.971	0.941	0.673
Heterogeneity on T2WI	0.500	0.875	0.139	0.475	0.556
Clarity of tumor margin	0.659	0.969	0.353	0.585	0.923
Tumor border shape	0.712	0.469	0.941	0.882	0.653
Tumor morphology	0.545	0.844	0.265	0.519	0.643
Location of unenhanced area	0.758	0.625	0.882	0.833	0.714
rDWI	0.606	0.969	0.265	0.554	0.900
cDWI_1500_	0.636	0.969	0.324	0.574	0.917
cDWI_2000_	0.700	0.938	0.471	0.625	0.889
cDWI_2500_	0.682	0.813	0.559	0.634	0.760
cDWI_3000_	0.742	0.719	0.765	0.742	0.743
cDWI_3500_	0.758	0.688	0.824	0.786	0.737
cDWI_4000_	0.742	0.656	0.824	0.778	0.718
Abnormal vaginal bleeding	0.561	0.313	0.764	0.588	0.551

*ACC* accuracy, *PPV* positive predictive value, *NPV* negative predictive value, *T2WI* T2-weighted image, *rDWI* real measured diffusion-weighted image with *b*-value of 1000 s/mm^2^, *cDWI*_*1500-4000*_ computed diffusion-weighted image (with *b*-values of 1500–4000 s/mm^2^)

### Measurement of ADC maps in ROIs

The mean areas of the ROIs on ADC maps were 508.86 mm^2^ (range 103.13–2259.38 mm^2^) for MMTU and 939.08 mm^2^ (range 106.25–4462.77 mm^2^) for T2HILM. The ROI area for the histogram analysis of the ADC maps was significantly larger in T2HILM than in MMTU (*p* = 0.036).

### Quantitative MRI characteristics of MMTU and T2HILM

In the comparison between MMTU and T2HILM, four MRI features, namely mean ADC value, skewness, kurtosis, and the largest diameter of the non-enhancing area, showed a significant difference (*p* < 0.05) (Table 5). ICC evaluation was excellent for tumor area and tumor diameter, moderate for the mean ADC value and diameter of the non-contrast area, and poor for skewness and kurtosis. The mean ADC values yielded moderate accuracy (AUC, 0.81; accuracy (ACC), 0.74) in differentiating between MMTU and T2HILM (Table 6). Similarly, the diameter of the non-enhancing area showed moderate accuracy (AUC, 0.77; ACC, 0.74), and skewness, kurtosis, tumor diameter, and tumor area showed low accuracy (Table 6).

**Table 5:** Quantitative MRI characteristics and a clinical parameter of T2 hyperintensity leiomyoma and MMTU

Characteristics	MMTU	T2HILM	ICC	*p* value
(Mean ± SD)	(*n* = 32)	(*n* = 34)		
Mean ADC value (× 10^−3^mm^2^/s)	1.029 ± 0.208	1.372 ± 0.947	0.688	< .0001^†^
Skewness	0.758 ± 0.634	0.404 ± 0.412	-0.0437	0.0078^†^
Kurtosis	4.597 ± 2.100	3.450 ± 0.911	0.0758	0.0039^†^
Diameter of tumor (mm)	116.63 ± 54.24	90.89 ± 53.96	0.906	0.571
Diameter of non-enhancing area (mm)	46.60 ± 49.21	11.39 ± 28.28	0.569	0.0004^†^
Area of tumor (mm^2^)	7409.32 ± 4885.64	5128.78 ± 4618.92	0.963	0.054
Age (year)	56.63 ± 9.18	49.85 ± 10.23	NA	0.006^†^

Values are given as mean ± standard deviation about characteristics of MMTU and T2HILM

*MMTU* malignant mesenchymal tumors of uterus, *T2HILM* T2 hyperintensity leiomyoma, *ICC* interclass correlation coefficients

^†^Significant difference between MMTU and T2 hyperintensity leiomyoma groups

**Table 6:** Statistical analyses of quantitative MRI characteristics and a clinical parameter

Characteristics	AUC	ACC	Sensitivity	Specificity	PPV	NPV	Cut off value
Mean ADC value	0.811^†^	0.742	0.813	0.677	0.703	0.793	1.187
Skewness	0.650	0.636	0.563	0.706	0.643	0.632	0.520
Kurtosis	0.653	0.652	0.344	0.941	0.846	0.604	5.041
Diameter of tumor	0.642	0.636	0.750	0.529	0.600	0.692	78.98
Diameter of non-enhancing area	0.766^†^	0.742	0.719	0.824	0.742	0.743	11.98
Area of tumor	0.661	0.667	0.594	0.735	0.679	0.658	6166.50
Age	0.705^†^	0.667	0.594	0.794	0.826	0.596	55.00

*AUC* area under the receiver operating characteristic curve, *ACC* accuracy, *PPV* positive predictive value, *NPV* negative predictive value

^†^AUC as moderate accuracy

The most discriminating mean ADC cutoff value to distinguish MMTU from T2HILM, as determined using ROC curve analysis, was 1.19 × 10^–3^ mm^2^/s. Similarly, the cutoff values for skewness, kurtosis, tumor diameter, non-contrast area diameter, area of the tumor, and age were 0.52, 5.04, 78.98 mm, 11.98 mm, 6166.50 mm^2^, and 55.00 years, respectively.

### Variant discriminant analysis

The 19 qualitative MRI findings, six MRI characteristics, and two clinical parameters were set as independent variables (Tables 3 and 5). A variant discriminant analysis was performed. In total, three selected findings with the corresponding coefficients and the constants are presented in Table 7. The remaining 24 findings were excluded because of low tolerance. A variable absent in the MRI findings, characteristics, and clinical parameters was set at 1; if it was present, the value was set at 2, and substituted by the following formula:

$$3.655x_{1} + 2.198x_{2} + 2.268x_{3} - 11.610$$

**Table 7:** Result of discriminant analysis

	Discrimination factor	*F* value	*p* value
T1 bright area	3.655	21.30	< .0001
cDWI_2000_	2.198	6.90	0.011
T2-hypointense band	2.268	5.93	0.018
discriminate threshold	− 11.610	NA	NA

*cDWI*_*2000*_ computed diffusion-weighted image (with *b*-values of 2000s/mm^2^)

where ${x}_{1}$= T1 bright area, ${x}_{2}$ = cDWI_2000_, ${x}_{3}$ = T2-hypointense band.

A negative discrimination score indicated T2HILM, and a positive discrimination score suggested MMTU. The classification accuracy of the variant discriminant analysis by combining the three selected findings of T1 bright area, cDWI_2000_, and T2-hypointense bands was 84.8% or 56/66 (T2HILM, 31/34; MMTU, 25/32), indicating high accuracy.

## Discussion

MRI has a good diagnostic ability to differentiate between benign and malignant tumors. Multiple MRI findings were used to distinguish between MMTU and T2HILM. However, a single MRI finding, such as T1 bright area, necrosis, or feather-like enhancement, has limited distinguishing ability and demonstrates relatively low accuracy compared to the combination of multiple findings [14, 23, 28, 29]. As a quantitative assessment method, the usefulness of the mean ADC value and the maximum tumor size has been reported; however, there was an overlap between MMTU and leiomyoma [8, 18, 23].

In our study, the presence of a T1 bright area, indicating subacute hemorrhage, had the highest accuracy (81.8%) as an independent single MRI and clinical finding to significantly distinguish MMTU from T2HILM. The overall sensitivity, specificity, positive predictive value (PPV), and negative predictive value (NPV) of the T1 bright area were 78.1%, 85.3%, 83.3%, and 89.6%, respectively. However, in a previous report by Kim et al. [9], the accuracy, sensitivity, specificity, PPV, and NPV of hemorrhage were 58.7%, 48.5%, 70.0%, 64.0%, and 55.3%, respectively, and there was no significant difference between MMTU and atypical leiomyoma. There are two possible reasons for this discrepancy. First, their MMTU included 55.5% endometrial stromal sarcomas compared to 37.5% in our study. Their report, which included a large number of low-grade endometrial stromal sarcomas that did not cause substantial bleeding, may have shown a lower accuracy than that observed in this study, which had a large percentage of high-grade endometrial stromal sarcoma, leiomyosarcoma, and undifferentiated uterine sarcoma. Second, Kim et al. did not differentiate according to the time of bleeding, whereas we separately examined subacute and chronic hemorrhages. Malignant tumors are known to hemorrhage repeatedly within the tumor and more often than benign tumors. However, subacute bleeding may not be common in T2HILM. These reasons may have resulted in a relatively good accuracy in our study.

In our study, the ADC value of MMTU was significantly lower than that of T2HILM. The decreased ADC values could be attributed to the restricted motion of water molecules [18, 30]. These results were similar to those of previous studies that reported lower ADC values in malignant sarcomas and endometrial stromal sarcomas than in benign leiomyomas [8, 18, 22, 23]. Furthermore, in this study, the ADC value showed the highest AUC (0.81) as a single quantitative parameter, which was slightly higher than the AUC of 0.74 reported for the combination of ADC value and DWI findings by Lin et al. [8], who compared the ADC values of leiomyosarcoma with those of ordinary leiomyoma. Ordinary leiomyomas show T2 blackout and yield low ADC values because of their fibrous components [18, 22]. We selected hyperintense leiomyomas on T2WI because malignant myometrial uterine tumors show high signal intensity. Therefore, our study showed a slightly higher AUC for differentiating between MMTU and T2HILM, although we only used mean ADC values.

Ordinary leiomyomas show signal homogeneity on T2WI [20], while MMTUs reveal signal heterogeneity [10]. Thomassin-Naggara et al. [22] showed that malignant uterine mesenchymal tumors were significantly more heterogeneous than benign leiomyomas, with accuracy, sensitivity, specificity, PPV, and NPV of 66.7%, 92.0%, 42.3%, 60.5%, and 84.6%, respectively. We expected that the heterogeneity on T2WI would be useful for distinguishing between MMTU and T2HILM; however, in our study, the accuracy, sensitivity, specificity, PPV, and NPV of heterogeneous signals were 50.0%, 87.5%, 13.9%, 47.5%, and 55.6%, respectively. Additionally, Lhakman et al. [14] used heterogeneity to differentiate between atypical leiomyomas and leiomyosarcomas; however, the accuracy (58.5%) was not high. The sensitivity, specificity, PPV, and NPV were 94.7%, 27.3%, 52.9%, and 85.7%, respectively. Fifty-seven point seven percent and 22.7% of the leiomyomas included by Thomassin-Naggara et al. and Lhakman et al., respectively, were non-T2HILM ordinal leiomyomas, whereas we did not include these. Therefore, heterogeneity on T2WI may have become less significant for differentiation between T2HILM and MMTU in our study.

Qiu Bi et al. [28] reported that abnormal vaginal bleeding is a predictive factor for differentiating uterine sarcoma from atypical leiomyoma. However, in our study, the presence of abnormal vaginal bleeding was not a significant differentiating factor between MMTU and T2HILM as a single parameter and in the multivariate analysis. In the study by Qiu Bi et al., uterine sarcomas were located predominantly in the uterine cavity in approximately half of the cases, whereas in our study, most MMTUs were located in the myometrium. Moreover, the percentage of atypical leiomyomas in the uterine cavity was only 5%, while the frequency of T2HILM cases in the uterine cavity was slightly higher at 12%. There are many causes of abnormal vaginal bleeding, including disruption of vulnerable blood vessels associated with tumors [31]. The difference in the frequency of the main locus of the tumor may have altered the rate of abnormal vaginal bleeding due to vascular disruption.

In this study, the use of cDWI with a high *b*-value (*b* = 1500–4000 s/mm^2^) showed higher accuracy in differentiating MMTU from T2HILM than the use of rDWI. The detection and diagnosis of solid malignant components can be improved using a high b-value DWI in the prostate and breast regions [32–34]. Furthermore, in ovarian tumors, Takeuchi et al. [35] showed that visual evaluation of cDWI_1500_ could distinguish decidualized endometriomas from ovarian cancers. This is because the higher the b-value, the greater the degree of signal attenuation from water molecules and the stronger the suppression of background normal tissues and other signals. However, the more cellular solid tumor area will continue to show a relatively high signal, accentuating the lesion [18]. In this study, there was no significant difference in discrimination ability when comparing b-values from 1500 to 4000 s/mm^2^. An excessively high b-value leads to diminished visualization and the appearance of background normal tissues, resulting in excessive loss of the signals of necrosis, cystic degeneration, and tumor. Furthermore, the sensitivity and NPV decreased, and specificity and PPV increased, with an increasing b-value. Thus, there may be a limit to the improvement in diagnostic performance that can be achieved by increasing the b-value.

The rational application of variant discriminant analysis to integrate different image findings is a statistically valid and logical method for maximizing diagnostic accuracy. Some new findings and characteristics were added while referring to previous reports, and 27 findings and characteristics were used as variables in our study. Subsequent analysis of the contribution of variables to the results using the stepwise method eliminated 24 findings and characteristics and established a reasonable model. The combination of the three criteria, namely T1 bright area, a high signal at cDWI_2000_, and T2-hypointense band, was the indicator with the highest discrimination (84.8%) for distinguishing MMTU from T2HILM.

Thomassin-Naggara et al. [22] found that the use of rDWI, mean ADC value, and T2 signal intensity in recursive partitioning model analysis achieved a diagnostic accuracy of 92.4% in distinguishing benign leiomyoma from MMTU. Qiu Bi et al. [28] proposed a model to differentiate uterine sarcoma from atypical leiomyoma, including T2HILM, using four features, e.g., abnormal genital bleeding, tumor located mainly in the uterine cavity, ill-defined tumor margins, and mean ADC value, and showed a high differentiation accuracy of 95.7%. Their accuracy was attributable to analyzing features including rDWI, mean ADC value, and T2 signal intensity. Abnormal genital bleeding, tumor location being mainly in the uterine cavity, ill-defined tumor margins, and the mean ADC value showed slightly higher discrimination ability than our accuracy based on the T1 bright area, T2-hypointense band, and cDWI_2000_. However, in their recursive partitioning model, T2 hypointense tumors were selected as leiomyomas, whereas in our study, T2 hypointense leiomyomas or T2 isointense leiomyomas were excluded from the beginning because it was problematic for T2HILM to differentiate between a malignant and benign uterine myometrium. Our model has a sufficiently high and useful diagnostic capability compared to those previously reported.

Our study had several limitations. First, it was retrospective, and our exclusive inclusion of patients with surgically resected tumors and preoperative MRI may have introduced selection bias. Although it was necessary to ensure an accurate pathological diagnosis, prospective studies are needed to confirm our results. Second, our sample size was small; therefore, a large-scale multicenter study consortium is needed in the future. Third, our study included images from multiple scanners with both 1.5-T and 3.0-T MR systems to compare MMTU and T2HILM. However, the three MRI findings of our variant discriminant analysis contained only qualitative analysis factors and no quantitative factors. However, our methods are widely applicable for diagnosing MMTU and T2HILM using both MR systems. Fourth, we did not compare the MRI findings with pathological findings. For example, T2 dark areas and T2-hypointense bands were distinguished from each other based on shape; however, the actual pathological findings were not confirmed. The imaging features of leiomyomas and MMTU identified in this study should be supported by radio-pathological correlation evaluations in future studies.

In conclusion, a T1 bright area on qualitative assessment and mean ADC value on quantitative assessment led to the most independent MRI differentiators of MMTU and T2HILM. Furthermore, variant discriminant analysis using the T1 bright area, cDWI_2000_, and T2-hypointense bands yielded high accuracy for differentiating between MMTU and T2HILM.
